# A Light-Driven Integrated Bio-Capacitor with Single Nano-Channel Modulation

**DOI:** 10.3390/nano12040592

**Published:** 2022-02-09

**Authors:** Jie Lin, Yu-Jia Lv, Lei Han, Kuan Sun, Yan Xiang, Xiao-Xing Xing, Yu-Tao Li

**Affiliations:** 1 College of Information Science and Technology, Beijing University of Chemical Technology, Beijing 100029, China; jaylin44@126.com (J.L.); 2020210529@mail.buct.edu.cn (L.H.); heaod1998@163.com (K.S.); 2 Beijing Key Laboratory of Bio-inspired Energy Materials and Devices, School of Space and Environment, Beihang University, Beijing 100191, China; lvyj16@buaa.edu.cn; 3 Beijing Institute of Nanoenergy and Nanosystems Chinese Academy of Sciences, Beijing 100083, China

**Keywords:** bacteriorhodopsin, photoelectric conversion, bioelectronics, nanopore, microfluidic

## Abstract

Bioelectronics, an emerging discipline formed by the biology and electronic information disciplines, has maintained a state of rapid development since its birth. Amongst the various functional bioelectronics materials, bacteriorhodopsin (bR), with its directional proton pump function and favorable structural stability properties, has drawn wide attention. The main contents of the paper are as follows: Inspired by the capacitive properties of natural protoplast cell membranes, a new bio-capacitor based on bR and artificial nanochannels was constructed. As a point of innovation, microfluidic chips were integrated into our device as an ion transport channel, which made the bio-capacitor more stable. Meanwhile, a single nanopore structure was integrated to improve the accuracy of the device structure. Experiments observed that the size of the nanopore affected the ion transmission rate. Consequently, by making the single nanopore’s size change, the photocurrent duration time (PDT) of bR was effectively regulated. By using this specific phenomenon, the original transient photocurrent was successfully transformed into a square-like wave.

## 1. Introduction

With the rapid development of bioelectronics [1,2,3,4,5,6,7], increasing numbers of bioelectronic devices have been developed and widely used. Bacteriorhodopsin (bR) extracted from *Halobacterium halobium* is a new type of biological material, which is widely used in bioelectronic devices. Moreover, bR is a novel photosynthetic system with a more controlled proton transport than chlorophyll in plants [8]. This consequently makes it easier to utilize in bioelectronic devices. The proton pump function of bR is the biophysical basis for designing novel bioelectronic devices. The pump function of bR would enable protons to be actively transported from the intracellular space to the extracellular under induced light [9,10]. Thus, an electrochemical potential difference inside and outside the cell membrane is formed, realizing photoelectric conversion [11,12,13,14,15,16,17,18]. Moreover, bR has many excellent material properties such as a higher light cycle stability [19], pH tolerance [20], thermal stability [19], and chemical stability [21], which improves its functional activity in the fabrication of bioelectronic devices outside the cell. Based on these material properties and the advantages of bR described above, it has been applied in a variety of bioelectronic devices, such as artificial retinal prosthesis [22], optical storage [23,24,25], photovoltaic cells [26], and pH sensors [27,28,29]. These applications are mainly based on the sensing, collection, and conversion of bR intrinsic photocurrent, while there is limited research on the shape regulation of photocurrent. The regulation of photocurrent shape can be achieved by some basic electronic components (e.g., capacitors), which greatly expands the application field of bR-based bioelectronic devices and lays a foundation for the construction of the next generation of intelligent bioelectronic systems.

As a typical electronic component, capacitors can easily output standard square waves with a controllable duty cycle through electronic circuits. A square wave can be used as the basic digital logic in a digital circuit, so it plays an important role in digital circuits [30]. However, in real life, most bio-photoelectric signals exist as a transient wave, resulting in limited applications. Therefore, transforming the transient wave to generate square waves by bio-capacitors will lead to the further development of bioelectronic digital logic and many related applications [31]. Some studies have used rhodopsin to fabricate biological capacitors to obtain waveform conversion functions. For example, Rao et al. used proteus rhodopsin and artificial anodized aluminum nanochannels to construct a light-powered bio-capacitor [32]; subsequently, they determined the influence of nanopore aperture on the photocurrent duration time (PDT) of the photocurrent of the bio-capacitor. However, the porous structure of the nanopore integrated into the device has a deviation from the size of nanopores. The maximum deviation could reach about 30% (171.6 ± 25.1 nm), which possibly makes the result inaccurate. An improved proposal is to replace the porous structure with a single-pore structure by novel nanopore fabrication technology. Meanwhile, in the bio-capacitor designed by Rao et al., the Ag/AgCl electrode used in the test system was unstable under induced light. Integrating the microfluidic chip may be a potential solution to enhance the device stability, and an electrode without light instability can make a device more stable. The design of these integrated devices is expected to improve the stability and accuracy of device performance and provide support for the performance improvement of bio-capacitors. 

In this work, microfluidic chip, bR, and Si/SiO_2_-based size-controllable Si_3_N_4_ single nanopore are combined to construct a light-driven bio-capacitor. After that, a test system is constructed to verify the capacitance properties of the entire system. It is found that the PDT is affected by the change in the nanopore size. Quantitative theoretical analysis based on the capacitor discharge process is proposed, as well as its capacitive property. Finally, this bio-capacitor is used to successfully convert the transient spike signal waveform of the bR into a square wave-like photocurrent waveform. This work is of great significance for the development of novel bR-based electronic components and the development of intelligent bioelectronics.

## 2. Materials and Methods

### 2.1. Modeling

**Figure 1:**
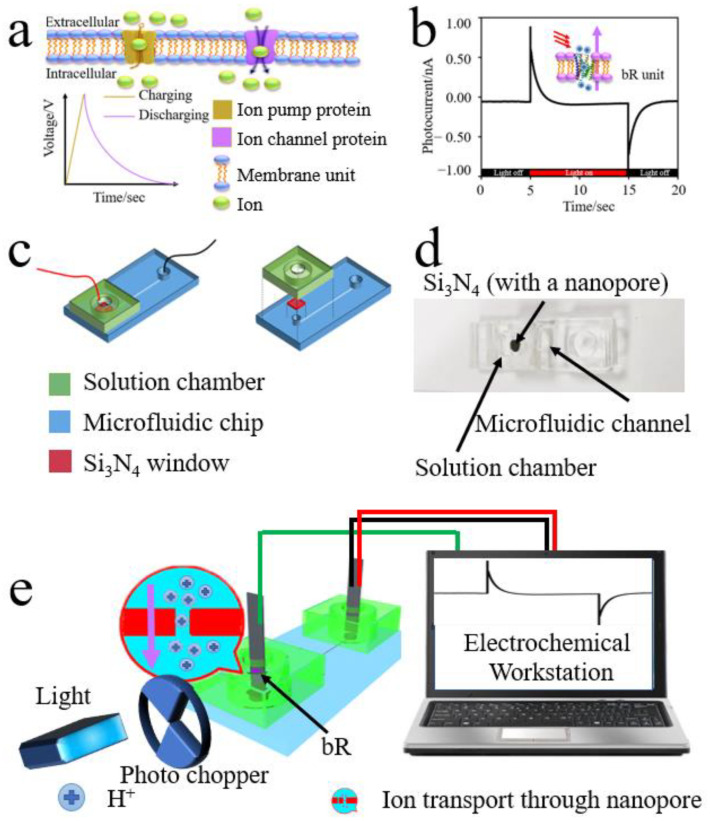
(**a**) The biological model of our device. The cooperation of ion-pump and ion-channel proteins makes the membrane act as a capacitor. (**b**) Photocurrent–time image of bR. (**c**) As-designed microfluidic chip. (**d**) Optical image of the device. (**e**) Schematic diagram of the complete test system.

Figure 1a shows a bionic schematic diagram of biological capacitance and it is the basis of the biological model of our device with capacitor property. This biological model consists of three parts: the membrane unit, ion pump protein (corresponding to the bR in our bio-capacitor), and the ion channel protein (corresponding to the nanopore in our bio-capacitor) [33,34,35]. Amongst them, the membrane unit (consisting of blue balls and yellow curves) is used to construct the purple membrane skeleton and support ion pump protein and ion channel protein. Ion pump proteins (yellow channel) can enable ions to reach the outside of the cell through active transport, thus forming a transmembrane electrochemical potential difference that is consistent with the function of bR. In addition, this process can simulate the charging process of the capacitor (represented by the yellow line in the voltage–time curve). The ion channel proteins (purple channel) and the nanopore structure in the device are functionally the same. They both can maintain the balance of ion concentration inside and outside the cell by transferring ions, exhibiting a discharging feature (represented by the purple line in the voltage–time curve). Therefore, nanopores can be analogous to ion channel proteins in the device. The cooperation of the ion pump protein (yellow channel) and the ion channel protein (purple channel) leads to the capacitance characteristic of the model. The purpose of the as-designed bio-capacitor is to simulate and regulate this biophysical process.

Figure 1b depicts a basic photocurrent curve of bR and it can be seen that the shape of the curve is in accordance with the charging process of the plasma membrane. Therefore, it can be inferred that bR can be used to simulate ion pump protein by designing a reasonable device structure. When the induced light is shining on bR, the retinal of bR is isomerized (from the all-trans form to the 13-cis form). Through this process, H^+^ ions are transferred from the intracellular to the extracellular, thereby generating potential differences between the inside and outside of the membrane. Based on this model and bR’s property, we use bR as the part of the device structure that acts as an ion pump protein, while the fabricated nanopore is used to act as an ion channel protein.

By designing the proper microfluidic chip, the nanopore can be integrated with the bR purple membrane into one device. Figure 1c displays the structure and fabrication process of the microfluidic chip, while Figure 1d demonstrates the optical image of the chip. The entire structure is on the left, and the individual structure is on the right. The entire chip is made up of three parts: the blue polydimethylsiloxane (PDMS) substrate, the red Si_3_N_4_ wafer with a nanopore, and the green solution chamber. Amongst them, the PDMS substrate contains a microfluidic channel (a semi-cylinder with a height of 1.8 cm and a radius of 162 μm), which links both sides of the solution to connect the outside electrode. During testing, 0.05 mol/L of Na_2_SO_4_ solution fills the microfluidic channel to make sure the ion can smoothly and stably transfer. The Si_3_N_4_ wafer with a nanopore (3 nm or 8 nm in diameter) can enable H^+^ to transfer from one side to the other to simulate the discharge process of ion channel proteins. The solution chamber has a hollow cylindrical structure (0.5 cm in diameter × 0.5 cm in height), which is used to store the Na_2_SO_4_ solution so that ITO glass with bR purple membrane can be in full contact with the solution. The entire microfluidic device is used to provide channels for H^+^ ion movement and support the Si_3_N_4_ window with good sealing. After the production of bio-capacitors, subsequent testing and analysis can be carried out.

During the test, we first connected various test equipment to the system (shown in Figure 1e). The inducing light provided by the light source (tungsten lamp PLX-SXE250) in the test system was 86,600 μW/cm^2^ and this was cut off by a photo chopper with a specific cutting frequency before shining on the bio-capacitor. A three-wire connection method was adopted to link the bio-capacitor with an electrochemical workstation. The green, yellow, and red lines in Figure 1e represent the working electrode, counter electrode, and reference electrode, respectively. Under the excitation by a flicker of light at a specific flickering frequency, bR transferred H^+^ ions from the intracellular to extracellular space and generated an electrochemical potential difference. Then, by controlling the size of the nanopore, the ion diffusion rate was controlled to reduce the potential difference. The photocurrent was collected by an electrochemical workstation (CHI660E, biasing condition is 0 V) and the photocurrent–time (I–t) curve was obtained. The connection mode of the electrochemical workstation was as follows: the working electrode was connected with the ITO electrode deposited with bR and the counter/reference electrode was connected with the pure ITO electrode.

### 2.2. Device Construction

**Figure 2:**
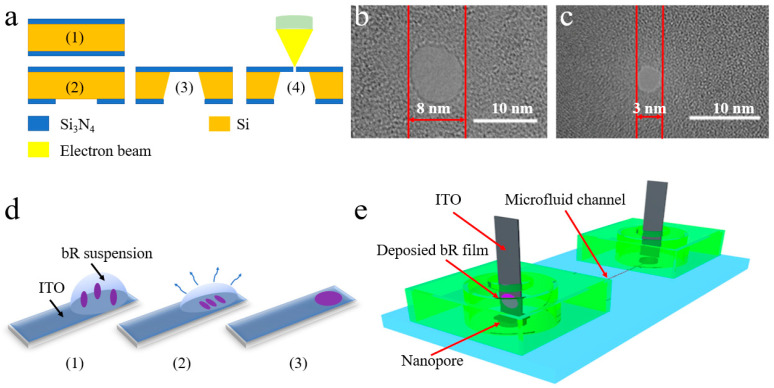
(**a**) Process flow of nanopore production. (**b**) TEM image of an 8 nm-size nanopore. (**c**) TEM image of 3 nm-size nanopore. (**d**) Process of evaporative deposition. (**e**) Complete device schematic. The PDMS substrate contains a microfluidic channel (a semi-cylinder with a height of 1.8 cm and a radius of 162 μm).

It should be noted that the nanopores used in the experiment have two different sizes: 3 nm or 8 nm diameter. This is of great significance for understanding the effect of nanopores size on device performance, in addition to improving the device accuracy. Figure 2a demonstrates the fabrication process used for nanopores: (1) deposition of Si_3_N_4_ film on both sides of the silicon wafer by low-pressure chemical vapor deposition; (2) the square area is lithographed and etched from the Si_3_N_4_ film on the back where the nanopore needs to be drilled; (3) the Si in the specific area is removed with wet anisotropic etching method; (4) a nano-scale hole in the Si_3_N_4_ film on the front is drilled with an electron beam. 

The TEM characterization [36] of two devices with different sizes of nanopores is shown in Figure 2b,c (the scale bar is 10 nm). Both holes appear to be quasi-circular. Two parallel red lines are used to highlight the size of the nanopore (8 nm on the left and 3 nm on the right). These two devices with different size nanopores are made to study the effect of the size in a subsequent test.

While there is a three-wire connection method in our test system, an ITO glass deposited with bR is used as a working electrode and the pure one is used as a counter/reference electrode. Figure 2d shows the following process: In step (1), 12 μL of bR suspension with a concentration of 1 mg/mL is dripped on the conductive side of ITO glass, which is then heated at 40 °C in step (2) to cause the excess solution to evaporate. Furthermore, step (3) demonstrates the modified electrode. 

The manufacturing process of the microfluidic chip with a nanopore can be divided into three parts: The first involves making the SU-8 mold, the second involves making the microfluidic chip, and the third involves integrating the PDMS and Si_3_N_4_ wafer. The specific steps are as follows:

1. SU-8 mold making:

(1) Prepare a clean silicon wafer with SU-8 photoresist spin-coating. Place the suspended silicon wafer on the homogenizer. (2) Transfer the static silicon wafer to a heating plate and then use a patterned glass chrome plate for UV exposure. (3) Transfer the exposed silicon wafer to a heating plate to stabilize the model. (4) Soak the silicon wafer in the developer for a while and then clean the remaining developer. (5) Heat the developed silicon wafer to harden the film. At this time, the SU-8 mold can be obtained.

2. Microfluidic chip production:

(1) Mix the PDMS and the supporting coagulant aid in a specific ratio then stir. (2) Place the processed container in the vacuum machine. (3) Use an aluminum paper box to keep the vacuumed PDMS in, flatten the mold to make sure that it is in full contact with the aluminum paper, then heat them. (4) Cut out the required cleaned model and perform ultrasonic cleaning. (5) Use the PLASMA CLEANER to treat the ultrasonically cleaned PDMS mold for viscous treatment. (6) Glue the mold components together and let them stand overnight.

3. Integration of PDMS and Si_3_N_4_ wafer:

Si_3_N_4_ wafer is bonded onto PDMS by the O_2_ plasma method. Turn the interface to be bonded upward and put it into the plasma cleaner machine (Harric PDC-002-HP), then work at 45 V power for one minute. After that, join the interface together, gently press, and leave it overnight.

Construction of the test platform: (1) Cut out 1.5 cm × 5 cm ITO glass with a glasscutter and have it cleaned. (2) Extract an appropriate amount of the bR solution, shake the solution evenly on the vortex oscillator. (3) Transfer the 12 μm bR solution to the ITO glass conductive surface. (4) Install in on the prepared microfluidic device. Subsequently, install the entire device into the shield box. (5) Place an opaque partition between the shielding box and the xenon light source. Thus, the whole test platform is constructed.

A complete schematic of the manufactured device is shown in Figure 2e, which shows the bR, the microfluidic channel, and the nanopores as well as their functions. The bR deposited in ITO is used as the working electrode in the test system and to simulate the ion pump protein. A microfluidic channel is used to transport H^+^ ions, while fabricated nanopores are integrated into the device upon the microfluidic channel to control the rate of diffusion of H^+^ ions.

## 3. Results

**Figure 3:**
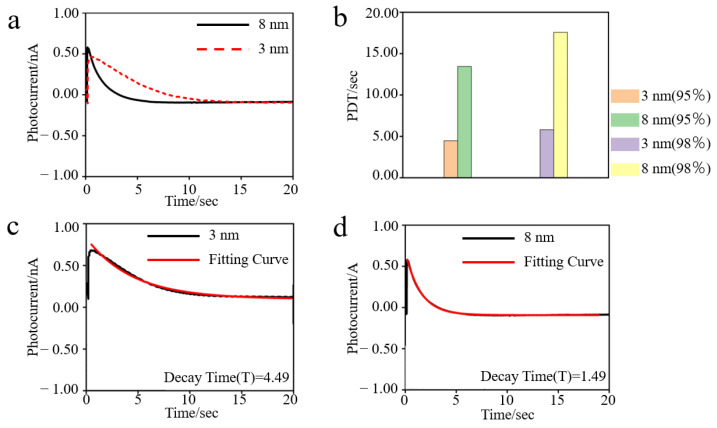
(**a**) The PDT is greatly affected by the changed nanopores. (**b**) The PDT in different indexes. (**c**) The fitting curve of 3 nm with a decay time of 4.49 s. (**d**) Fitting curve of 8 nm with a decay time of 1.49 s.

**Figure 4:**
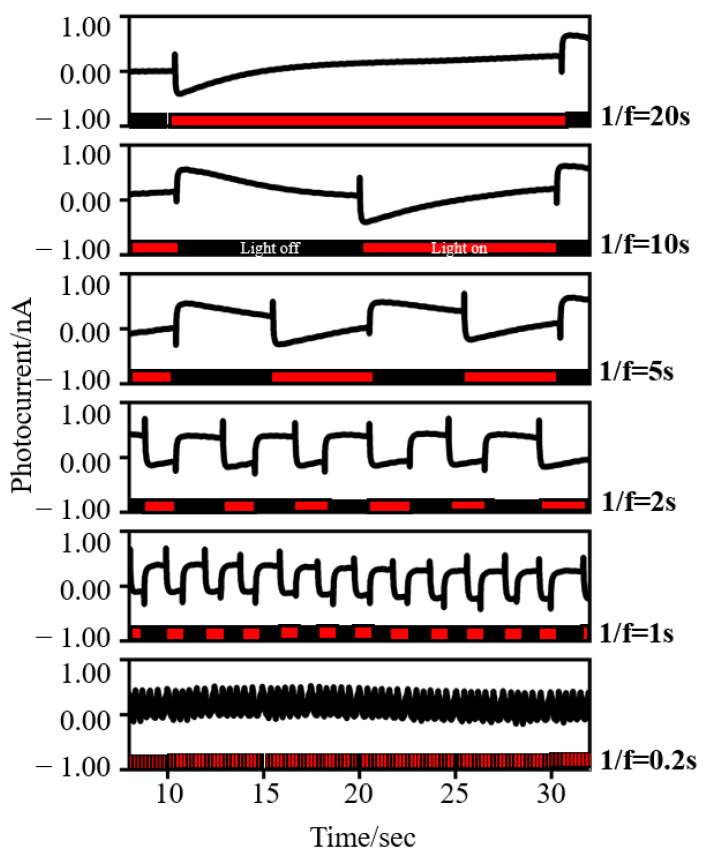
The photocurrent waveform changes with the light flickering frequency. f represents the flickering frequency of the induced light.

Figure 3a demonstrates the comparison of two different fabricated devices’ photocurrent curves. It is clear that the device integrated with 3 nm nanopores has a longer sustainable photocurrent duration than another one (integrated with 8 nm nanopores). To make the quantitative difference more intuitive, photocurrent duration time (PDT) is defined and shown in Figure 3b. Figure 4 demonstrates that the photocurrent has been successfully converted.

PDT [32] contains the light cycle time of bR and the decay time of the photocurrent. The decay time of the photocurrent reaches the seconds level, which is much longer than the light cycle time of bR (microsecond level). Thus, PDT could be simply defined as the decay time of the photocurrent. The decay time of the photocurrent shows a descending tendency in the i–t image; hence, an exponential decay function is used to fit the line. In addition, the time parameter (T) of the function is used to evaluate the PDT, which is defined as the time when the fitting line descends 95 percent. This is demonstrated by the following function:

Y = Y_0_ + Ae^−X/T^

where Y represents the instantaneous photocurrent, Y_0_ represents the initial photocurrent, A represents the amplitude of photocurrent, X represents the X-axis (time), and T represents the decay time. By this equation, the data from the peak photocurrent amplitude down to 5% photocurrent amplitude (the photocurrent amplitude descends 95 percent) are selected for the fitting calculation as T. Then, the decay time (T) is obtained and PDT is calculated. Figure 3c,d demonstrate that the fitting result is in accordance with the experimental results.

## 4. Discussion

**Figure 5:**
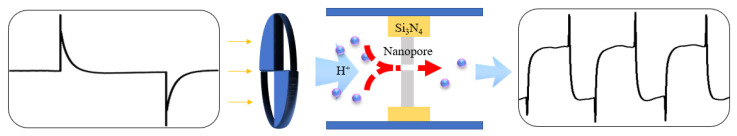
Schematic diagram of the mechanism underlying the photocurrent.

Figure 5 illuminates the mechanism underlying the photocurrent. First, since the ion pump function of bR protein can transport H^+^ ions to the extracellular (EC) side, there will be an ion concentration gradient on both sides of the nanopore. Then, the concentration gradient descent rate is regulated by the size of the nanopore. H^+^ ions passively diffuse to the other side through the nanopore, and this charge movement process forms a current. The size of the nanopore will influence the amount of H^+^ ions passing through the pore per unit time, affecting the equilibrium time of the potential difference at both sides. Then, the PDT becomes larger under a smaller nanopore size, resulting in a slowly descending photocurrent. Controlling the light flickering frequency causes the chopping to occur in a period where the photocurrent changes are relatively flat, meaning that the photocurrent waveform can be adjusted and converted from peak signal to square wave signal.

It can be seen from Figure 3 that the smaller the nanopore is, the longer the PDT is, which represents a longer charging and discharging time in the bio-capacitors under a different process parameter. The decay times (that is, PDT) of different nanopore sizes are 4.49 s and 1.49 s, corresponding to 3 nm and 8 nm, respectively. As shown in Figure 3, the PDT of a 3 nm device is about 3 times that of the 8 nm device. Compared with the previous data [32], they both show the same linear relationship—that is, the PDT becomes longer as the size of the nanopore decreases.

Since this study was conducted under a low frequency condition, bR has sufficient time to complete its whole photocycle process [37]. Therefore, in our experiment, the amplitude of the photocurrent varies little under different light flickering frequency, which is also confirmed by previous studies [32].

Based on bR’s specific phenomenon, we believe that the PDT can also be regulated by the light flickering frequency. Through this, the bio-capacitor’s charging and discharging time can be regulated. As shown in Figure 4, the transient spike current waveform can be successfully converted into a square wave-like waveform with the change in the light flickering frequency. In the bio-capacitor system, the photocurrent response changes with the increase in the light flickering frequency; the main characteristic of this is the descending tendency of the photocurrent to be flat. When the light flickering frequency reaches 0.5 Hz, the transient spike waveform is transformed into a square wave-like waveform. Theoretically, this phenomenon will lead to a longer PDT, verifying the successful regulation of the charging and discharging time of a build-up bio-capacitor. 

The other potential application for regulating the photocurrent is that the square waveform can be seen everywhere in digital electronic circuits and expressed as binary “0” and “1” through analog-to-digital conversion. Simultaneously, because the bio-capacitor can form a square-like waveform while the light is on, a selective filter can be made by controlling the frequency of flickering illumination.

## 5. Conclusions

In this paper, the design and construction of a bR bio-photoelectric system is carried out and a bR-based bio-capacitor system is built. The photocurrent–time curve of this bio-capacitor system is measured in the electrochemical workstation and analyzed. The system with 3 nm-diameter nanopores has a PDT of about 13.45 s, while another device with 8 nm-diameter nanopores has one of about 4.47 s. Obviously, the PDT of the bR is regulated by the changing of the size of the nanopore channel. The i–t curve proves that bR has bio-capacitor properties. Finally, through optimizing the control parameters and the light flickering frequency, the original transient spike photocurrent waveform of the bR can be successfully converted into a square wave signal that is more suitable for the combination of micro-device circuits. As a newly developed bio-device, the nano-channel modulated bio-capacitor has shown great potential in the application of biological devices in the future.

## Data Availability

The data presented in this study are available on request from the corresponding author. The data are not publicly available due to privacy concerns.
